# Fibrous bands associated with higher Masaoka stage and poor recurrence‐free survival in patients with thymoma

**DOI:** 10.1111/1759-7714.13755

**Published:** 2020-11-25

**Authors:** Kazuhiro Minami, Naoe Jimbo, Yugo Tanaka, Takahiro Uchida, Takeshi Okamoto, Nahoko Shimizu, Takefumi Doi, Daisuke Hokka, Tomoo Itoh, Yoshimasa Maniwa

**Affiliations:** ^1^ Division of Thoracic Surgery Kobe University Graduate School of Medicine Kobe Japan; ^2^ Department of Diagnostic Pathology Kobe University Graduate School of Medicine Kobe Japan

**Keywords:** Fibrous bands, hematoxylin and eosin‐stained slide, Masaoka stage, recurrence‐free survival, thymoma

## Abstract

**Background:**

Fibrous bands (FBs) are one of the histological features in tumors which can be confirmed by hematoxylin and eosin (H&E)‐stained slides. FBs have been reported to correlate with malignancy in various tumors. This study aimed to investigate whether the presence of FBs is associated with malignancy in thymoma.

**Methods:**

A total of 123 consecutive patients with thymoma who underwent microscopically complete resections from January 2000 to December 2018 were enrolled into this study. H&E‐stained slides of all thymoma patients were re‐examined. Study patients were classified into two groups: with FBs (*n* = 36) and without FBs (*n* = 87). Clinicopathological characteristics, overall survival (OS), and recurrence‐free survival (RFS) were compared between the two groups. Furthermore, multivariate analyses were performed to identify whether the presence of FBs was associated with higher Masaoka stage and poor prognosis in patients with thymoma.

**Results:**

The Masaoka stage was found to be higher and recurrence more likely in thymoma patients with FBs than in those without. RFS was significantly poorer in thymoma patients with FBs than in those without, although no significant difference was observed in OS between them. The presence of FBs was significantly associated with higher Masaoka stage in the multivariate analysis using logistic regression. Additionally, the presence of FBs was an independent prognostic factor for poor RFS in multivariate analysis using Cox's proportional hazards model.

**Conclusions:**

The presence of FBs in patients with thymoma was associated with higher Masaoka stage, higher recurrence rate, and poorer RFS.

**Key points:**

**Significant findings of the study:**

Fibrous bands (FBs) are bands of fibrosis dividing tumors into different‐sized irregular islands. The presence of FBs is associated with higher Masaoka stage and poor recurrence‐free survival in patients with thymoma.

**What this study adds:**

The presence of fibrous bands might be associated with the malignant behavior of thymoma. Confirming the presence or absence of FBs may result in personalized medication for patients with thymoma.

## Graphical abstract

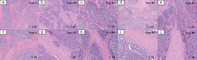

• The presence of FBs is associated with the malignant behavior of thymomas.

• Confirmation of FBs might lead to individualized medications in patients with thymoma.

## Introduction

Thymoma is a rare tumor that originates from the epithelial cells of the thymus with an incidence of 0.13 per 100 000 person‐years and exhibits a spectrum of low‐ to high‐grade malignancy.^1^ According to the World Health Organization (WHO) classification system, thymoma is broadly classified into two categories based on morphological characteristics of tumor cells: type A, including spindle epithelial cells, and type B, including epithelioid/polygonal cells. Further categorization is based on lymphoid infiltration rates: types B1, B2, and B3 thymomas, respectively. Type A thymomas with lymphoid infiltration are categorized as type AB thymomas^2^, ^3^ ^3^; they are considered to exhibit low‐grade malignancy, whereas type B thymomas are considered to exhibit moderate‐ to high‐grade malignancy.^4^, ^5^, ^6^, ^7^, ^8^, ^9^ Treatment options for thymomas include surgery, chemotherapy, and radiation therapy. Among these, surgical resection is considered the best treatment choice with curative intent.^10^, ^11^, ^12^

With regard to the postoperative prognosis of patients with thymoma, various studies have demonstrated an association between WHO histological subtypes and its prognosis.^4^, ^5^, ^6^, ^7^, ^8^, ^9^ However, recurrence or metastasis sometimes occurs even in patients considered to have low‐grade malignancy according to WHO histological subtypes.^13^, ^14^, ^15^, ^16^, ^17^ Therefore, other histological factors that might estimate the prognosis of patients with thymoma should be identified.

In the current study, we focused on histological features of fibrous bands (FBs), ie, bands of fibrosis dividing tumors into different‐sized irregular islands. Several studies have suggested that the histological feature of FBs might be correlated with malignancy in various tumors.^15^, ^18^, ^19^, ^20^, ^21^, ^22^, ^23^, ^24^

For these reasons, we hypothesized that the histological feature of FBs might be associated with malignancy in thymoma. To address the hypothesis, all types of completely resected thymomas were carefully histologically analyzed.

## Methods

### Patient population

From January 2000 to December 2018, 131 consecutive patients with thymoma underwent surgical resection at Kobe University Hospital. Among them, patients who did not achieve microscopically complete resections (R1, 2) and those who underwent preoperative therapies, including induction chemotherapy, steroid therapy, and radiation therapy, were excluded from this study because these preoperative therapies would raise challenges for an accurate pathological diagnosis and may influence the expression of FBs. Finally, 123 patients with thymoma who achieved microscopically complete resections were included (R0). Surgical procedures were mainly thymothymectomies, but extended thymectomies were performed in patients with thymoma with myasthenia gravis (MG). This study was approved by the Ethics Committee of Kobe University Hospital (No. B200047).

### Histopathology

Histological diagnoses of thymoma were based on hematoxylin and eosin (H&E) staining results and WHO classification system criteria: types A, AB, B1, B2, and B3.^2^ The Masaoka staging system was used to determine the clinicopathological staging, because several studies on thymoma have been previously described based on this staging system.^10^ All patients were diagnosed by two or three pathologists from our hospital.

### Definition of FBs

**Figure 1:**
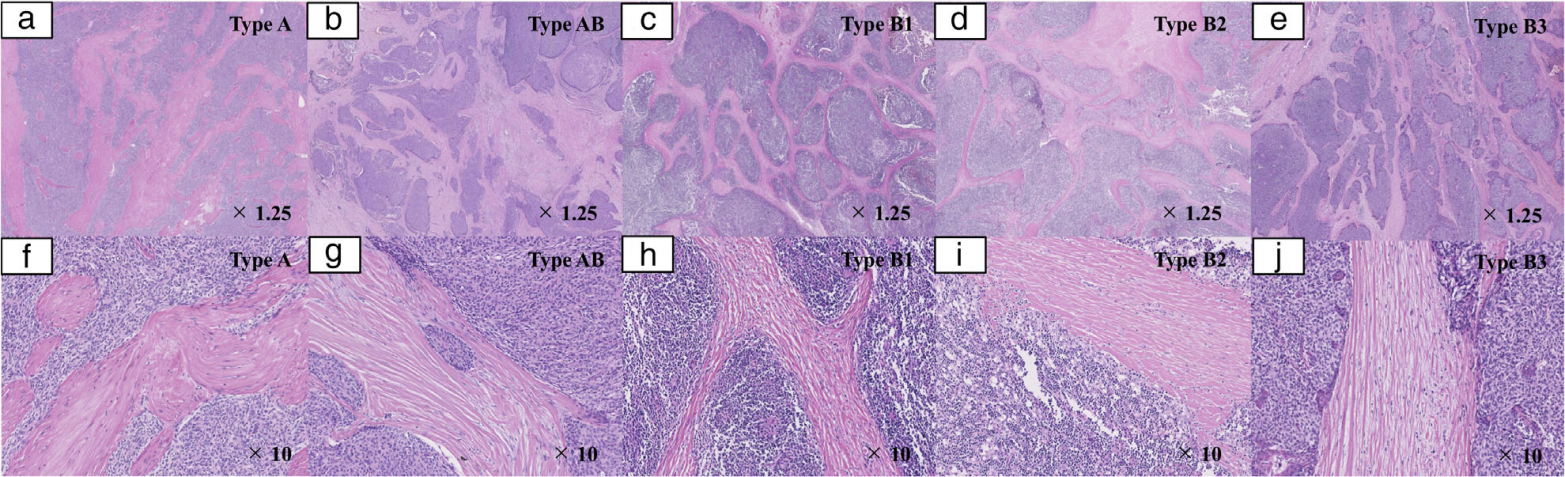
Representative pathological images of thymoma patients with FBs. FBs dividing tumors into different‐sized irregular islands were thick enough to be observed at low magnification (×1.25). FBs contained small amounts of fibroblasts and lymphocytes but rarely contained tumor cells. Hyalinosis was observed (×10). (**a**) Type A thymoma (×1.25); (**b**) type AB thymoma (×1.25); (**c**) type B1 thymoma (×1.25); (**d**) type B2 thymoma (×1.25); (**e**) type B3 thymoma (×1.25); (**f**) type A thymoma (×10); (**g**) type AB thymoma (×10); (**h**) type B1 thymoma (×10); (**i**) type B2 thymoma (×10); (**j**) type B3 thymoma (×10). FBs, fibrous bands.

**Figure 2:**
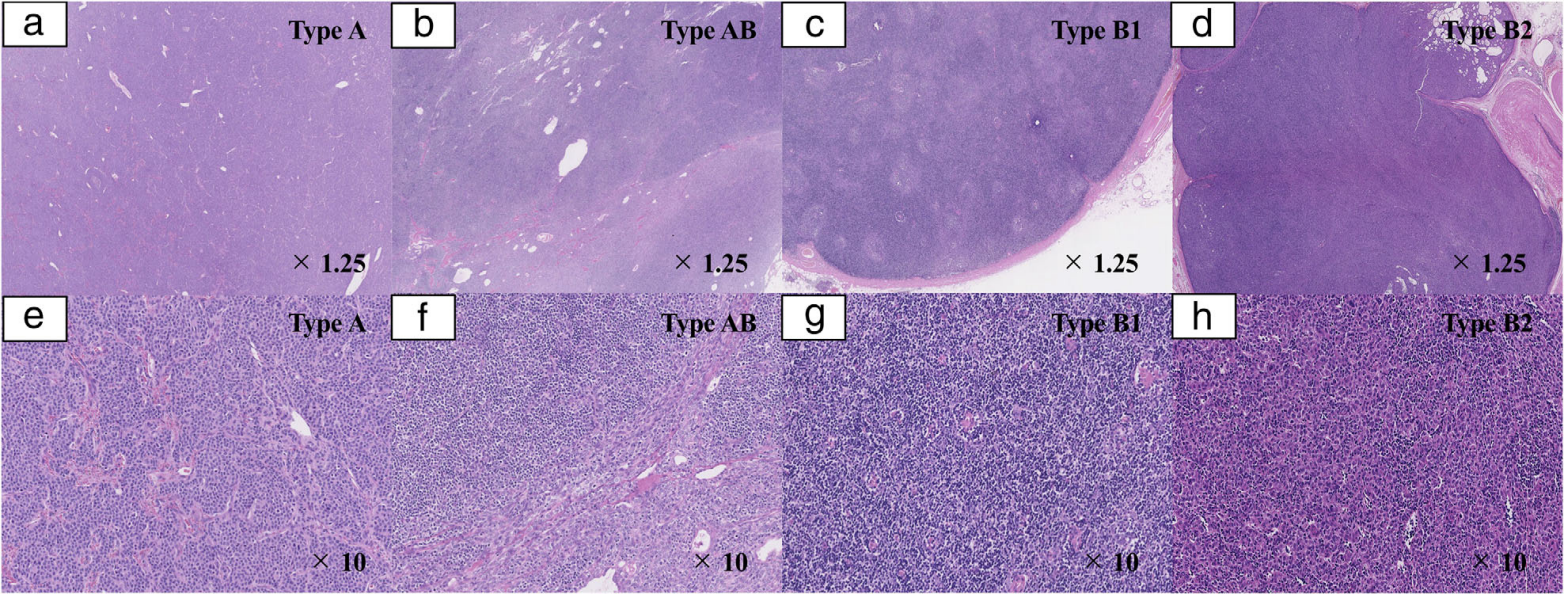
Representative pathological images in thymoma patients without FBs. FBs were not observed in these patients. Images of type B3 thymoma without FBs are not shown because all patients with type B3 thymoma had FBs. (**a**) Type A thymoma (×1.25); (**b**) type AB thymoma (×1.25); (**c**) type B1 thymoma (×1.25); (**d**) type B2 thymoma (×1.25); (**e**) type A thymoma (×10); (**f**) type AB thymoma (×10); (**g**) type B1 thymoma (×10); (**h**) type B2 thymoma (×10). FBs, fibrous bands.

H&E‐stained slides of all patients with thymoma were re‐examined. In this study, FBs were defined as FBs based on the following histological factors: (i) FBs divided tumors into different‐sized irregular islands; (ii) FBs containing small amounts of fibroblasts and lymphocytes, but rarely containing tumor cells; (iii) presence of hyalinosis; and (iv) width of FBs larger than some tumor islands and thick enough to be observed at low magnification. If FBs did not fulfill the definition mentioned above, such FBs were not regarded as FBs. We evaluated more than three H&E‐stained slides, including those of the sections of the largest cross‐sectional slice. If the presence of FBs was confirmed even in one slide, the patient was considered to have FBs. Patients with FBs were defined as “thymomas with FBs” and those without FBs as “thymoma without FBs.” Figs 1 and 2 showed the representative pathological findings of thymomas with and without FBs, respectively.

### Study patients

Patients with thymoma were divided into two groups: those with FBs and without FBs. Clinicopathological characteristics, overall survival (OS), and recurrence‐free survival (RFS) were compared between the two groups. Finally, multivariate analyses were compared to identify whether the presence of FBs was associated with higher Masaoka stage and with poor prognosis in patients with thymoma.

Clinical information, including age, sex, tumor diameter, Masaoka stage, WHO classification, surgical procedure, presence of MG, and outcomes were retrieved from the medical records. OS was defined as the time from the date of operation to death from any cause or last follow‐up visit. RFS was defined as the time from the date of operation to disease relapse or death from any cause.

### Statistical analysis

JMP version 13 software (SAS Institute, Cary, NC, USA) was used to perform all statistical analyses. Differences in patient characteristics between groups were evaluated by analysis of variance, chi‐square or Fisher's exact test. The OS and RFS were evaluated by Kaplan–Meier survival analysis, and the log‐rank test was used to evaluate differences in distributions. Factors for predicting higher Masaoka stage were assessed by performing multivariate analyses using logistic regression. Prognostic factors for predicting postoperative survival were assessed by performing multivariate analyses using Cox's proportional hazards model. *P*‐values of <0.05 were considered to indicate statistical significance.

## Results

### Clinicopathological characteristics of patients with thymoma

Table 1 summarizes the clinicopathological characteristics of patients with thymoma. A total of 123 patients with thymoma were included in this study, comprising 68 (55%) male and 55 (45%) female patients, with an average age of 59.0 (range: 20–84) years. The average tumor diameter was 51.8 (range: 8–150) mm. Among them, 50 had stage I (41%), 56 had stage II (45%), 16 had stage III (13%), and one had stage IV (1%). Moreover, 12 patients had type A thymoma (10%), 34 had type AB thymoma (28%), 34 had type B1 thymoma (28%), 41 had type B2 thymoma (33%), and two had type B3 thymoma (1%). A total of 43 patients (35%) underwent extended thymectomies and 80 (65%) underwent thymothymectomies. All patients in this study underwent microscopically complete resections (R0). A total of 44 patients (35%) had MG complications. There were 10 patients (8%) postoperative recurrence, and four (3%) died during follow‐up.

**Table 1:** Clinicopathological characteristics among thymoma patients with or without FBs

Characteristics	All patients(*n* = 123)	With FBs (*n* = 36)	Without FBs (*n* = 87)	*P*‐value
Age (years)				0.76
Mean	59.0	59.5	58.7	
Range	20–84	27–82	20–84	
Sex				0.87
Male	68 (55)	19 (53)	49 (57)	
Female	55 (45)	17 (47)	38 (43)	
Tumor diameter (mm)				0.51
Mean	51.8	56.4	49.9	
Range	8–150	16–110	8–150	
				0.22
≥50 mm	56 (46)	20 (56)	36 (41)	
<50 mm	67 (54)	16 (44)	51 (59)	
Masaoka stage				< 0.0001
I	50 (41)	7 (19)	43 (49)	
II	56 (45)	14 (39)	42 (49)	
III	16 (13)	14 (39)	2 (2)	
IV	1 (1)	1 (3)	0 (0)	
				<0.0001
I/II	106 (86)	21 (58)	85 (98)	
III/IV	17 (14)	15 (42)	2 (2)	
WHO histological subtypes				0.10
A	12 (10)	4 (11)	8 (9)	
AB	34 (28)	7 (19)	27 (31)	
B1	34 (28)	6 (17)	28 (32)	
B2	41 (33)	17 (47)	24 (28)	
B3	2 (1)	2 (6)	0 (0)	
				0.42
A/AB	46 (37)	11 (31)	35 (40)	
B1/B2/B3	77 (63)	25 (69)	52 (60)	
Surgical procedure			26 (30)	0.10
Extended thymectomy	43 (35)	17 (47)		
Thymothymectomy	80 (65)	19 (52)	61 (70)	
Presence of MG	44 (35)	17 (47)	27 (31)	0.13
Recurrence	10 (8)	10 (28)	0 (0)	<0.0001
Mortality	4 (3)	3 (8)	1 (1)	0.053

Values are presented as *n* (%) or mean.

FBs, fibrous bands; MG, myasthenia gravis; WHO, World Health Organization.

### Clinicopathological findings of thymoma patients with or without FBs

Table 1 also summarizes the clinicopathological characteristics of thymoma patients with or without FBs. Among all patients with thymoma in this study, 36 (29%) had FBs and 87 (71%) did not have FBs. No significant differences in age, sex, tumor diameter, WHO histological subtypes, surgical procedure, presence of MG, and mortality rate were observed between the groups (*P* > 0.05), but significant differences in the Masaoka stage and recurrence rate were observed (*P* < 0.0001, and *P* < 0.0001, respectively). Table S1 showed clinicopathological characteristics of patients with thymoma who relapsed postoperatively. All 10 patients who relapsed postoperatively were confirmed to have FBs. Eight patients had Masaoka stage III diseases and two had Masaoka stage I diseases. One patient had type AB thymoma, two had type B1 thymoma, six had type B2 thymoma, and one had type B3 thymoma. None of the thymoma patients without FBs had relapsed postoperatively.

### Comparison of clinical outcomes in thymoma patients with or without FBs

**Figure 3:**
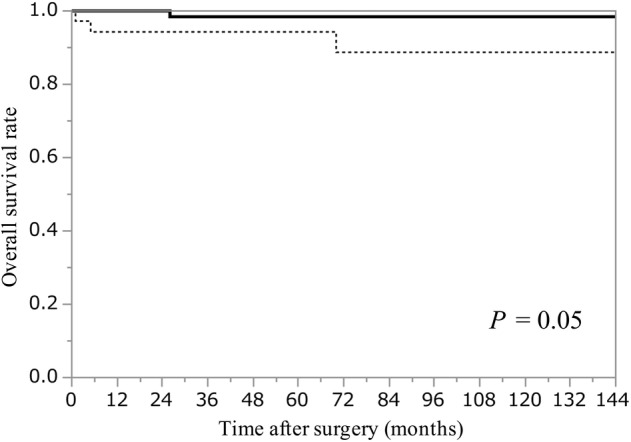
Kaplan–Meier curves of the overall survival rate in thymoma patients with or without FBs. FBs, fibrous bands; 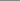, Without FBs (n = 87); 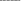, With FBs (n = 36).

**Figure 4:**
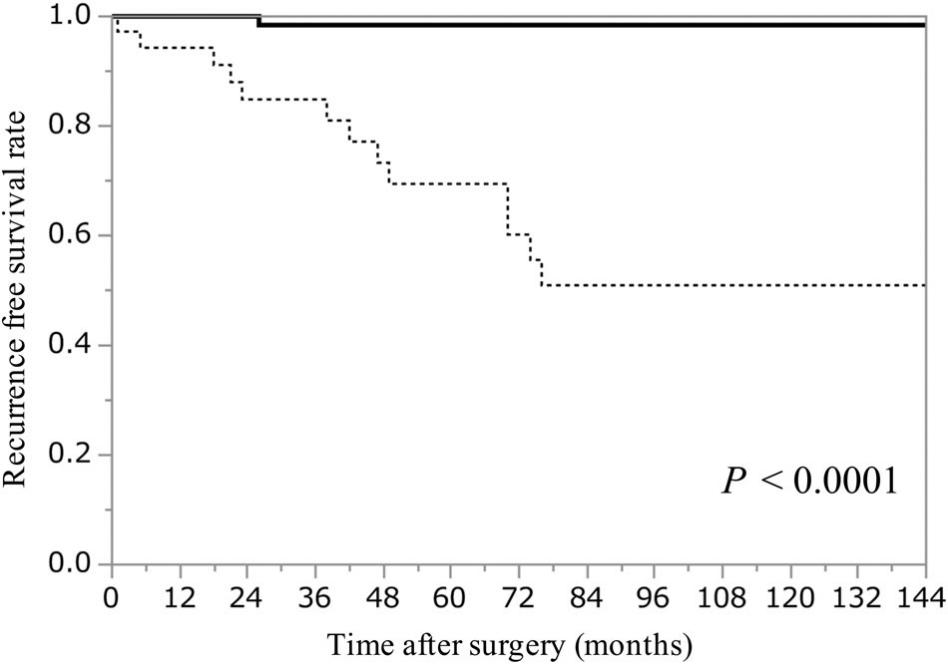
Kaplan–Meier curves of recurrence‐free survival rate in thymoma patients with or without FBs. FBs, fibrous bands; 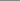, Without FBs (n = 87); 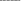, With FBs (n = 36).

The five‐ and 10‐year OS rates were 94% and 89%, respectively in thymoma patients with FBs and 98% and 98% in thymoma patients without FBs, respectively. No significant difference in OS was observed between the groups (*P* = 0.05; Fig 3). The five‐ and 10‐year RFS rates were 69% and 51% in thymoma patients with FBs, respectively and 98% and 98% in thymoma patients without FBs, respectively. RFS was significantly poorer in thymoma patients with FBs than that in patients without FBs (*P* < 0.0001; Fig 4).

### Multivariate analysis of factors associated with higher Masaoka stage and poorer RFS

To identify factors influencing the higher Masaoka stage, multivariate analysis including five variables considered to influence higher Masaoka stage (age, sex, tumor diameter, histology, and FBs) was performed. Table 2 presents the multivariate analysis results. Although age, sex, tumor diameter, and histology were not significantly associated with higher Masaoka stage, the presence of FBs was significantly associated with higher Masaoka stage (Table 2, odds ratio, 31.32; 95% confidence interval [CI]: 6.308–155.5; *P* < 0.0001).

**Table 2:** Multivariate analysis of factors influencing the higher Masaoka stage

Variable	OR	95% CI	*P*‐value
Age			
≥70 vs. <70 years	2.423	0.611–9.605	0.21
Sex			
Male vs. female	1.315	0.350–4.937	0.69
Tumor diameter			
≥50 vs. <50 years	2.337	0.620–8.812	0.21
Histology			
A, AB vs. B1, B2, B3	0.329	0.073–1.487	0.15
FBs			
Present vs. absent	31.32	6.308–155.5	<0.0001

CI, confidence interval; FBs, fibrous bands; OR, odds ratio.

Next, to identify prognostic factors influencing the RFS, multivariate analysis including five variables considered to influence the RFS (age, sex, Masaoka stage, histology, and FBs) was performed. The multivariate analysis results are shown in Table 3. Patients with Masaoka stages I and II thymoma had significantly better RFS than those with Masaoka stages III and IV thymoma (Table 3, hazard ratio, 0.088; 95% CI: 0.015–0.328; *P* = 0.0001). Thymoma patients with FBs had significantly poorer RFS than thymoma patients without FBs (Table 3, hazards ratio, 7.996; 95% CI: 1.030–165.2; *P* = 0.046).

**Table 3:** Multivariate analysis of prognostic factors influencing RFS

Variable	HR	95% CI	*P*‐value
Age			
≥70 vs. <70 years	1.867	0.594–5.636	0.27
Sex			
Male vs. female	1.520	0.518–4.729	0.45
Masaoka stage			
I, II vs. III, IV	0.087	0.015–0.328	0.0001
Histology			
A, AB vs. B1, B2, B3	0.713	0.107–2.834	0.66
FBs			
Present vs. absent	7.996	1.030–165.2	0.046

CI, confidence interval; FBs, fibrous bands; HR, hazard ratio; RFS, recurrence‐free survival.

## Discussion

In this study, the presence of FBs was found to be associated with higher Masaoka stage, higher recurrence rate, and poorer RFS in patients with thymoma. Moreover, the presence of FBs was an independent factor for higher Masaoka stage and poor RFS in patients with thymoma. These results supported our hypothesis that the presence of FBs might be associated with malignancy in thymomas.

The prognosis of patients with thymoma after surgical resections has been previously reported to be dependent mainly on the following three factors: resection status (R0, 1, or 2), Masaoka stage, and WHO histological subtypes.^4^, ^9^, ^12^, ^25^ Among them, resection status has been considered to be strongly correlated with the prognosis.^9^, ^25^ Therefore, we limited the current study to patients in which microscopically complete resections (R0) were achieved to eliminate its impact on the prognosis, with the intention that the association between FBs and prognosis would be more obvious.

Regarding WHO histological subtypes, the prognosis of patients with thymoma has been considered to be worse in types A, AB, B1, B2, and B3, respectively; types A and AB thymomas have been regarded as low‐grade malignancy and types B1–B3 have been regarded as moderate‐ to high‐grade malignancy.^4^, ^5^, ^6^, ^7^, ^8^, ^9^ However, recurrence or metastasis still occurs, even in type A and AB thymomas, which have been considered as low‐grade malignancy,^13^, ^14^, ^15^, ^16^, ^17^ and other histological factors that might estimate the prognosis of patients with thymoma should be determined. Our data showed that the presence or absence of FBs might be correlated with the prognosis of patients with thymoma. Therefore, the presence or absence of FBs might be better confirmed along with confirming the Masaoka stage and WHO histological subtypes, when estimating the prognosis of patients with thymoma.

A possible association between the histological feature of fibrosis and their malignancy has been described in various tumors.^18^, ^19^, ^20^, ^21^, ^22^, ^23^, ^24^ For example, the presence of FBs has been reportedly associated with poor prognosis in patients with lung squamous cell carcinoma.^18^ Similar reports have been well described in the field of colorectal cancer research.^21^, ^22^, ^24^ In thymoma research, only one study mentioned the association between histological feature of FBs and thymoma malignancy, as reported by Vladislav *et al*. who investigated 37 patients with type AB thymomas.^15^ They reported the prognostic value of two architectural patterns in type AB thymomas. One histological feature of architectural patterns is the presence or absence of FBs. They reported that type AB thymomas with FBs were associated with higher Masaoka stage at diagnosis with a greater likelihood of recurrence.^15^ Our current study differs from that of the report by Vladislav *et al*. because we conducted survival and multivariate analyses in all histological thymoma subtypes. To our knowledge, this study might be first to show the presence of FBs associated with malignancy in all types of thymomas.

Whether thymomas with FBs show malignant behaviors remains to be elucidated. The presence of FBs is considered a result of epithelial–mesenchymal transition (EMT). EMT is the process of transforming epithelial cells into mesenchymal‐like cells such as fibroblasts, losing their cell polarity, and cell adhesion capacity. In tumor progression, this process is associated with fibroid morphology, tumor invasion, and metastasis.^18^, ^24^, ^26^, ^27^, ^28^, ^29^ Thymomas also appear to gain their malignant behavior through EMT, which is characterized by the loss of E‐cadherin.^30^, ^31^, ^32^ For these reasons, thymomas showing the histological feature of FBs might allow invasive and metastatic capacity through EMT. However, no studies have reported an association between the histological feature of FBs and EMT in thymomas. Further studies based on molecular biology are needed to verify the association.

In clinical practice, our study findings might provide new insights in order to establish treatment strategies for patients with thymoma. First, none of the thymoma patients without FBs showed recurrence postoperatively, even if they had been found to have advanced disease. This result was consistent with the study by Vladislav *et al*.^5^ and should be highly noted. All types of thymomas may possibly recur and metastasize, even if they are type A and AB thymomas, which are considered as low‐grade malignancy,^13^, ^15^, ^16^, ^33^, ^34^ and patients where recurrence is unlikely might be identified by just confirming H&E‐stained slides. Moreover, adjuvant therapies such as chemotherapy or radiation therapy might be indicated to determine the presence or absence of FBs. Second, two thymoma patients with FBs who had Masaoka stage I diseases showed recurrences, indicating that thymoma patients with FBs should be carefully followed postoperatively, even in their early Masaoka stages. Third, the EMT process might be a new therapeutic target for thymomas. For example, tumor‐associated fibroblasts have been previously reported to play an important role in EMT.^28^, ^35^ The fact that tumor‐associated fibroblasts contribute to the fibroid morphology of tumors and tumor progression is strongly supported by several studies,^18^, ^36^, ^37^, ^38^, ^39^, ^40^ and tumor‐associated fibroblasts might be a potential therapeutic target, especially for invasive thymomas.

Confirming the presence or absence of FBs in thymomas is very simple and easy; it relies only on H&E staining information and can be performed at any facility without any special immunostaining. These results might lead to personalized medication for patients with thymoma in the future.

This study has several limitations. First, this was a retrospective study with a small number of patients and included only those who had surgically resected thymomas. A larger scale study including nonsurgical cases is needed to verify our results. Second, the fundamental question why the presence of FBs is associated with malignancy in a molecular level was not answered. Therefore, further studies should investigate how molecular properties of thymoma cells contribute to fibroid morphology and tumor aggressiveness in thymomas.

In conclusion, our results indicated that the presence of FBs in patients with thymoma was associated with higher Masaoka stage, higher recurrence rate, and poorer RFS. We hope that our findings will help provide these patients with new treatment options. Multicenter studies with large sample sizes and molecular‐based researches are needed to verify our results in the future.

## Disclosure

The authors declare that they have no conflicts of interest related to this study.

## Supporting information


**Table S1** Clinicopathological characteristics of patients with thymoma who relapsed after a microscopic complete resection.
