# Supplementary material for: Fibrous bands associated with higher Masaoka stage and poor recurrence‐free survival in patients with thymoma
Source: Thorac Cancer. 2020 Nov 25;12(3):349–56. doi: 10.1111/1759-7714.13755 (PMC7862782; doi:10.1111/1759-7714.13755)
Supplement: Supplementary file 1 — Table S1 Clinicopathological characteristics of patients with thymoma who relapsed after a microscopic complete resection. [file TCA-12-349-s001.docx]

| **Supplementary table** | | | | | | | | |
| --- | --- | --- | --- | --- | --- | --- | --- | --- |
| Clinicopathological characteristics of patients with thymoma who relapsed after a microscopic complete resection. | | | | | | | | |
|  |  |  |  |  |  |  |  |  |
| Case | Age (years) | Sex | Tumor diameter (mm) | FBs | Masaoka stage | WHO histological classification | Time to relapse (months) | OS  (months) |
|  |  |  |  |  |  |  |  |  |
|  |  |  |  |  |  |  |  |  |
| 1 | 40 | M | 60 | + | III | B2 | 76 | 193 |
| 2 | 75 | F | 65 | + | III | B3 | 74 | 83 |
| 3 | 59 | F | 44 | + | I | B1 | 70 | 85 |
| 4 | 70 | M | 70 | + | III | B2 | 49 | 81 |
| 5 | 75 | M | 40 | + | III | AB | 38 | 67 |
| 6 | 60 | F | 49 | + | I | B2 | 42 | 55 |
| 7 | 67 | M | 35 | + | III | B2 | 47 | 54 |
| 8 | 61 | F | 85 | + | III | B1 | 21 | 43 |
| 9 | 67 | M | 85 | + | III | B2 | 18 | 35 |
| 10 | 39 | F | 50 | + | III | B2 | 23 | 109 |
| M: Male, F: Female, FBs: Fibrous bands, OS: Overall survival, +: Present, WHO: World Health Organization. | | | | | | | | |
|  |  |  |  |  |  |  |  |  |
